# Vaccination status of patients using anti-TNF therapy and the physicians’ behavior shaping the phenomenon: Mixed-methods approach

**DOI:** 10.1371/journal.pone.0223594

**Published:** 2019-10-04

**Authors:** Hussain Abdulrahman Al-Omar, Hadeel Magdy Sherif, Ahmed Yaccob Mayet

**Affiliations:** Department of Clinical Pharmacy, College of Pharmacy, King Saud University, Riyadh, Saudi Arabia; University of Wisconsin Madison School of Pharmacy, UNITED STATES

## Abstract

**Objective:**

Anti-tumor necrosis factor (Anti-TNF) therapy improves the prognosis and reduces the morbidity and mortality associated with many chronic inflammatory autoimmune diseases. However, as it is linked to an increased infection risk, appropriate vaccination is required. The study aimed at investigating the vaccination status of patients receiving Anti-TNF therapy and physicians’ perceptions of and views about vaccinating these patients.

**Methods:**

A sequential explanatory mixed-methods approach was used. The study comprised a quantitative, retrospective drug utilization review for determining institutional consumption of Anti-TNF therapy and an assessment of vaccination status in patients prescribed Anti-TNF therapy to audit physicians’ adherence to Anti-TNF therapy-related vaccination recommendations. Patient data from electronic medical records (EMRs) obtained from tertiary care hospitals between September 2015 and September 2017 were used. Further, a qualitative study using a phenomenographic approach with semi-structured interviews of 12 physicians was carried out to explore the physicians’ perceptions, views, and recommendations of vaccinating patients who are undergoing Anti-TNF therapy and identifying factors that may cause poor adherence to vaccination recommendations.

**Results:**

Forty-three of 310 patients receiving Anti-TNF therapy were vaccinated. Infliximab was the most frequently prescribed agent, accounting for 96.7% of total orders. Eight of the 12 physicians stated that they were aware of vaccination guidelines and seven viewed pre–Anti-TNF therapy vaccination as essential because of the high infection risk and claimed to incorporate it in their daily practice. Barriers to adherence included ignorance of recommendations, workload, vaccine unavailability, and advanced disease state.

**Conclusion:**

Although the recommendations published by professional medical societies emphasized the importance of vaccination before initiating Anti-TNF therapy, few patients were vaccinated. Medical administration in hospitals should develop policies, procedures, and guidelines for vaccination; implement education programs for physicians and patients and procure vaccines in a timely way to improve their use.

## Introduction

Tumor necrosis factor α (TNFα) is a biomolecule that regulates innate immunity. Its dysfunction leads to acute inflammation, apoptosis, and cellular proliferation. TNFα role has been well-established in the etiology of rheumatoid arthritis (RA), inflammatory bowel disease (IBD), psoriasis, plaque psoriasis, Behçet’s disease, sarcoidosis, and ankylosing spondylitis [1]. This finding has led to the prompt development of Anti-TNFα therapies such as infliximab, etanercept, adalimumab, golimumab, and certolizumab. These agents target the inflammatory process and promote marked clinical remission, improve quality of life, and reduce morbidity and mortality even in patients with an inadequate response to conventional treatment. Furthermore, these therapies are well tolerated, can prevent disease progression, and, in many circumstances, have been shown to reverse damage to the target organ in different disorders [1–8].

Despite all the reported benefits associated with the use of Anti-TNF therapy, [9–13] it poses an increased risk of infections such as pneumococcal pneumonia, meningococcal meningitis, seasonal influenza, and hepatitis B viral (HBV) infection that can be prevented by vaccination before initiating Anti-TNF therapy [14–17]. The risk of infections can be further aggravated by using immunosuppressant drugs in the therapeutic regimen [18]. In literature, mortality due to pneumococcal pneumonia and HBV infection has been reported in patients treated with biologic therapy [19, 20]. A meta-analysis carried out in the United States (US) concluded that the use of Anti-TNF therapy increased the chance of the occurrence of any infection by 20% and serious infections by 40% [15]. Results from the US national data for Healthcare Cost and Utilization Project in 2008 showed the cost associated with a serious infection, including the cost of hospitalization and an antibacterial regimen, was US$20,781 per infection [21]. Another study reported that the highest cost associated with hospitalization among RA patients was due to adalimumab and methotrexate (US$475.21), followed by infliximab and methotrexate (US$354.91), and etanercept (US$232.62). The use of adalimumab alone was associated with the lowest adverse effect-related cost (US$122.96) [22]. The costs associated with serious infections and physician visits in these studies can be minimized, if not completely avoided, by the use of vaccines. As a result, global drug regulatory agencies have warned healthcare providers of the risk of viral, bacterial, and fungal infections associated with Anti-TNF therapy. Moreover, several recommendations and guidelines have been published to emphasize the importance of vaccination before initiating Anti-TNF therapy. These recommendations identify the types of vaccines, either live-attenuated or inactivated, that can be used during and before Anti-TNF therapy and the appropriate timing of vaccination before starting Anti-TNF therapy [7, 23–29].

A few quantitative studies have addressed physicians’ adherence to vaccination recommendations while prescribing Anti-TNF therapy on specific diseases, and most concluded that the use of vaccines was suboptimal with considerable variation in vaccinations rates and type [30–39]. Furthermore, there is a lack of studies that examine such concerns in Saudi Arabia. It should be noted that, until the time of writing this article, there were no qualitative or mixed-methods studies that explore the behavior of physicians who prescribing Anti-TNF therapy. The use of mixed-methods helped not only to identify the extent of the problem but also to explore and explain the behavior behind the phenomenon under investigation thoroughly. In mixed-methods, the use of a quantitative approach can provide a general understanding of the research problem and ensures the generalization of the results, whereas the use of a qualitative approach can provide a rich, contextualized understanding of some aspect of human experience through the intensive study of a particular phenomenon. When used in combination, quantitative and qualitative methods complement each other and allow for more robust analysis, taking advantage of the strengths of each. Therefore, the aim of our study, using a mixed-methods approach, was to focus on the vaccination status of patients receiving Anti-TNF therapy, physicians’ adherence to the vaccination recommendations, and physicians’ perceptions of and views about vaccination before prescribing Anti-TNF therapy.

## Methods

The study was conducted in a tertiary care and referral teaching hospital in Saudi Arabia by using a sequential explanatory mixed-methods approach comprising a retrospective quantitative study followed by a qualitative phenomenographic study of physicians prescribing Anti-TNF therapy. The reason for choosing the sequential explanatory mixed-methods approach, a two-phase approach composed of an initial quantitative study followed by a qualitative study to examine and explain unexpected findings in research, is that it allowed us to harness the benefit of the pragmatic perspective inherent in the mixed-methods approach. This design permitted us to identify the vaccination status for patients on Anti-TNFs therapy from the quantitative data, and to study and generate an in-depth understanding of the complex phenomenon of physicians’ prescribing practice of vaccines for patients receiving Anti-TNF therapy in its natural setting in Saudi Arabia using qualitative data. These benefits cannot be cultivated by using the quantitative or the qualitative methods alone.

The quantitative study consisted of patients’ data extracted from the hospital’s electronic medical records (EMRs) for the period from September 2015 to September 2017. The data were obtained electronically for the required variables and manually (including prescriber name, prescriber professional position, department, and timing of the vaccination initiation) for incomplete electronically extracted variables to ensure data completeness. The final dataset included the following variables of interest: patient age, gender, diagnosis, co-morbidities, vaccination status, vaccines administered, the timing of any vaccination initiation, and infection risk; Anti-TNF therapy used, dose, dosage form, frequency, and prescriber name; and prescriber’s professional position and department. Classification of the patients based on the risk of infection was obtained from the pre-existing classification provided by the EMRs. The data were imported to Microsoft Excel^®^, revised for duplicate entries, and merged into a master file.

The qualitative study consisted of in-depth semi-structured interviews using a validated interview topic guide comprising 10 open-ended questions, which was fully developed and validated by the authors and can be found in S1 File to capture in-depth physicians’ perceptions of and views about Anti-TNF therapy prescription and adherence to published vaccination recommendations in addition to the identification of barriers that may influence it. Physicians who were prescribing Anti-TNFs therapy to adult patients for approved indications in Saudi Arabia were invited to participate in the study. Physicians who did not have the prescribing privilege of Anti-TNFs were excluded from the study. Purposeful and snowball sampling techniques were applied to recruit physicians who prescribed Anti-TNF therapy with different specialties, ages, genders, and years of experience, to maximize variations and diversity and achieve data saturation. On the day of the interviews, the study’s aim, objectives, and interview process were explained to physicians before conducting the interviews. Signed consents were obtained from participating physicians for recording interviews and publishing findings. All interviews were digitally recorded and then transcribed verbatim. Notes were taken manually and incorporated in the analysis with the transcripts. Interviews and physicians’ demographics, which included name, professional position, age, gender, department, years of practice, and nationality, were then imported to NVivo^®^ Version 11 to facilitate data coding and sorting.

For the quantitative study, descriptive statistics, frequency, percentages, mean, and standard deviation (SD) were calculated. Inferential statistics, using Pearson’s Chi-square test, were calculated to examine the association between vaccination status and the risk of infection. The Bonferroni correction for multiple comparisons was used to reduce the chance of Type I error. Statistical significance was defined as a *p*-value of <0.05. The analysis was performed using IBM SPSS^®^ Version 24.0 (Armonk, NY: IBM Corporation, 2016).

For the phenomenographic study, the seven-step approach for phenomenographic analysis proposed by Dahlgren et al. was followed to obtain the categories of description of the perceptions and views about using vaccines with Anti-TNF therapy [40]. The six steps of thematic analysis proposed by Braun et al. were also used to explore the barriers that affect physicians’ adherence to vaccination recommendations [41]. The analyses were performed by HA and HS independently and then the findings were discussed to determine the final categories of description, themes, and subthemes. The findings were then presented using tables and figures displaying the four main categories of description, main themes and subthemes, supporting quotes, and outcome space.

Ethical approval for this research was granted by King Saud University Medical City (KSUMC) institutional review board (IRB; IRB number: E17-2499). All patients’ data that were obtained from the hospital EMRs were fully anonymized before they were accessed by the authors. No informed consent from the patients was required by the IRB committee since the data released was secondary anonymized data.

## Results

**Fig 1:**
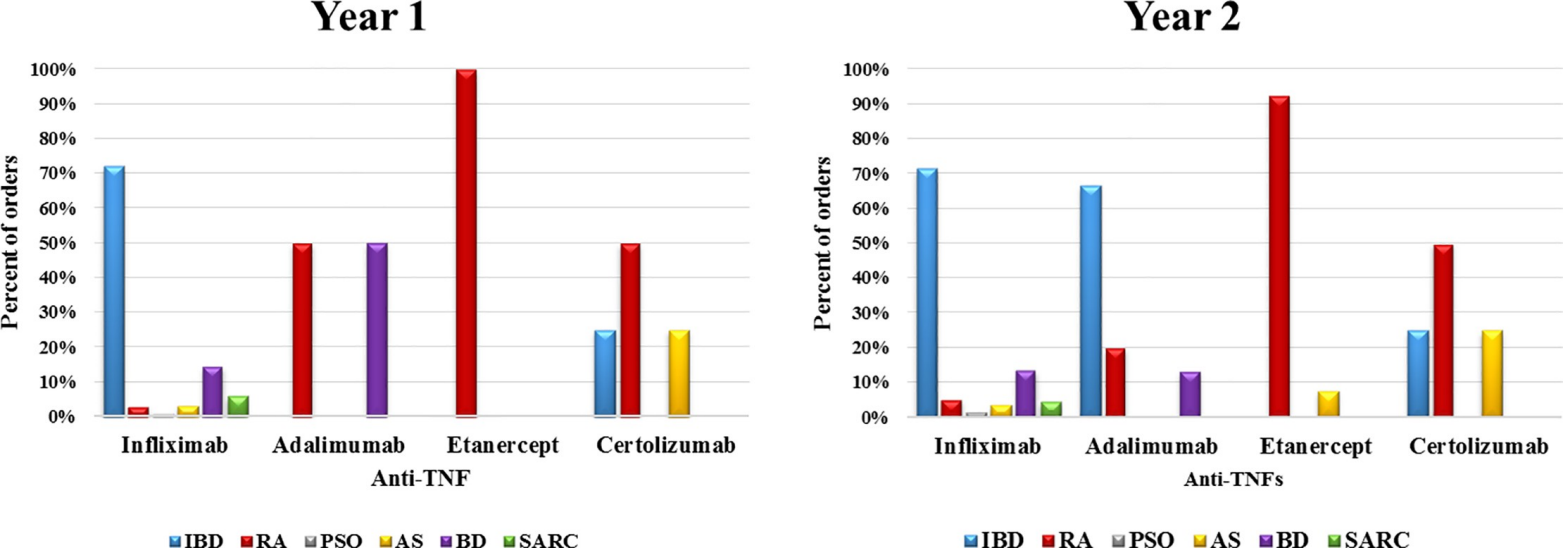
DUR of Anti-TNFs by indication during first and second years at KSUMC. IBD: inflammatory bowel disease; RA: rheumatoid arthritis; PSO: psoriasis; AS: ankylosing spondylitis; BD: Behcet’s disease; SARC: Sarcoidosis.

We identified 310 patients receiving Anti-TNF therapy during the study period; their mean age was 33.6 ± 13.08 years. Among these patients, 126 (40.6%) were aged between 18 and 27 years, and 169 (54.5%) were male. Infliximab was found to be the most frequently prescribed Anti-TNF therapy, accounting for 97.2% of the total hospital orders in the first year of the study and 96.5% in the second, as shown in Table 1. The most common diagnosis for which Anti-TNF therapy was prescribed in the hospital was IBD (65%) as demonstrated in Fig 1.

**Table 1:** DUR and Sales of Anti-TNFs during year 1 and year 2.

Year	Anti-TNF	Hospital Orders
**1**^**st**^	Infliximab	528
	Adalimumab	10
	Certolizumab	--
	Etanercept	5
**2**^**nd**^	Infliximab	1,578
	Adalimumab	38
	Certolizumab	5
	Etanercept	14

Overall, only 43 (13.9%) patients received some of the recommended vaccines, while 71% (220) were identified as being at a high risk of infection. Vaccination status was not significantly associated with the risk of infection classification (*p* = 0.75). With regards to the timing of vaccine administration, 35 of 69 patients (50.7%) received vaccines before Anti-TNF therapy. Among the recommended vaccines, influenza, hepatitis B, and pneumococcal vaccines were ordered for 31 (55.4%), 9 (16.1%), and 11 (19.6%) of 56 patients, respectively. Gastroenterologists were the medical professionals who most commonly prescribed vaccines, with 24 (55.8%) as shown in Table 2.

**Table 2:** Results of institutional vaccines prescribing by physicians in patients receiving Anti-TNFs.

	Number (n)	Percentage (%)
***Vaccination status (n = 310)***		
Not vaccinated	267	86.1
Vaccinated	43	13.9
***Risk of infection(n = 310)***		
Patients without risk of infection	90	29.0
Patients with risk of infection	220	71.0
***Vaccination status of those with a risk of infection (n = 221)***		
Not vaccinated	187	85.0
Vaccinated	33	15.0
***Departments (n = 43)***		
Gastroenterology	24	55.8
Pulmonology	13	30.2
Rheumatology	6	14.0
***Vaccine orders (n = 56)***		
Hepatitis B adult vaccine	9	16.1
Influenza virus vaccine inactivated	31	55.4
Meningococcal vaccine	1	1.8
Pneumococcal vaccine	11	19.6
Varicella virus vaccine	4	7.1
***Vaccine order timing (n = 69)***		
After Anti-TNF administration	3	4.3
Before Anti-TNF administration	35	50.7
During Anti-TNF administration	31	44.9

In the qualitative study, 12 physicians were required to reach data saturation; Table 3 shows the physicians’ demographic characteristics. The findings from the phenomenographic analysis revealed four main categories of description: awareness, adherence, practice, and perception. Each category has three main views within it. Table 4 shows each category and the corresponding views and supporting quotations. Among the 12 physicians, 8 stated that they were aware of the vaccination guidelines, and seven viewed vaccination before Anti-TNF therapy as an essential step for the high risk of infection. Among these seven, only the dermatologists did not claim to apply this belief to their practice. Six physicians acknowledged that they did not adhere to the recommendations.

**Table 3:** Demographics of the participating physicians in interviews.

Code	Age	Gender	Years of experience
P1	42	Male	17
P2	40	Male	17
P3	51	Female	25
P4	48	Female	20
P5	43	Male	22
P6	53	Male	25
P7	60	Male	30
P8	37	Male	15
P9	50	Male	29
P10	45	Male	16
P11	34	Male	7
P12	50	Male	22

**Table 4:** Findings of the phenomenographic analysis with regards to variation in physicians’ views and perceptions about vaccination for patients receiving Anti-TNF.

Category of description	Variation in behavior	Total number of physicians	Example supporting quote
**Awareness**	Aware	8	*“The EULAR and the ACL Yes*, *both of them do (have vaccination recommendations)*.*” (P3)*
	Unfamiliar with guidelines in their specialty	2	*“In dermatology*, *there are no (vaccination) guidelines*, *but when we go to the rheumatology*, *GI*, *because of most of their diseases*, *they are immunocompromized by say*. *It's not like our disease*, *and we don't consider psoriasis patient as an immunocompromized patient*. *But when you look to the studies Inflammatory Bowel Disease and Rheumatoid Arthritis*, *they advise them to take certain vaccine*.*” (P6)*
	Complete lack of awareness about any guidelines	2	*“No*, *I'm not aware (of any vaccination recommendations*.*)” (P8)*
**Adherence**	Adhere	2	*“For the last two years*, *I think all of us are more of adherence to vaccination*.*” (P4)*
	Not declared	4	*“Yeah*, *so there are recent*, *I think there are recent recommendations materials have been given*, *provided by our pharmacy department at the hospital*. *We have our clinical pharmacist; Dr*. *W I guess*. *So*, *she has also provided us with some guidelines for that*.*” (P9)*
	Don’t Adhere	6	*“Especially on Anti-TNF or most of the biologics*, *I need to initiate (vaccines) as I said*, *but I'm not doing it very frankly speaking*, *I'm not able to do it because of these technical difficulties*.*” (P3)*
**Practice**	Vaccinate all patients	7	*“It's before starting Anti-TNFs*, *so anybody about to start anti-TNF (will receive vaccines)*.*” (P12)*
	Vaccinate certain patients	2	*“I think it has something to do with patient’s related risk factors*.*” (P2)*
	Don’t vaccinate	3	*“As I told you*, *I'm not vaccinating*.*” (P1)*
**Perception**	Vaccines are not always needed	2	*“Personally*, *I don't vaccinate my patients*. *I never vaccinate any patient*, *especially patient who's actually negative for certain infection…I think it has something to do with patient’s related risk factors*.*” (P2)*
	Vaccines are always needed	7	*“I think it's better to have a vaccinated patient because we know that biologics predispose patients to infection sometimes*.*” (P7)*
	Concerned about timing and safety	3	*“Yes*, *the (timing of vaccination given to Anti-TNF patients) I think it should be put as a part of the guidelines or the policies*, *especially in our hospital*. *All of us know that*, *yes*, *we know not to give the vaccinations at least two weeks prior (to Anti-TNF initiation)*, *but the problem I face myself is to time the (vaccines)*.*” (P3)*

**Fig 2:**
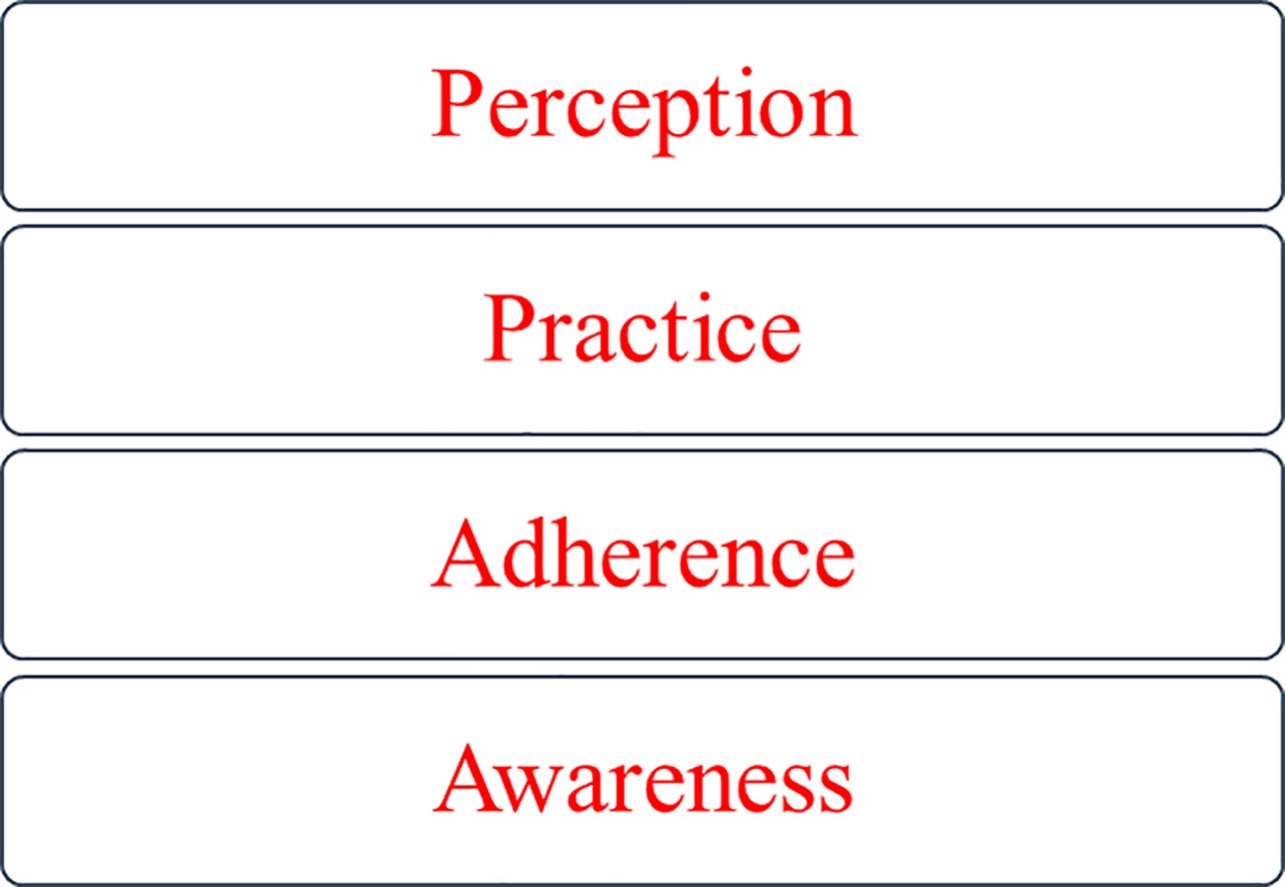
Outcome Space arranged from the least complex dimension from the bottom the most complex at the top.

We arranged the categories of description in a hierarchy, as shown in Fig 2, with increasing complexity as the levels moved to the top. Awareness was found to be the least complex since it involved the interaction of the physicians with the recommendations. Adherence came next, as it entailed more complexity than awareness. It was followed by practice, which was placed at a higher-level still due to the fact that it encompassed the complex interaction between physicians, patients, the healthcare system, and the recommendations. Perception was put at the highest level, the most complex, since it is affected by more general aspects.

The thematic analysis, which explored the barriers that hindered adherence to vaccination recommendations, revealed three main themes—physician-related barriers, patient-related barriers, and healthcare-system-related barriers—each of which contained subthemes. Table 5 summarizes these themes and the 14 subthemes with supporting quotations. In the thematic analysis, we identified the following main barriers for the use of vaccines: lack of physicians’ knowledge, patients’ advanced disease state, lack of policy implementation, physicians’ workload, and vaccine unavailability.

**Table 5:** Findings of the Thematic Analysis regarding factors that hinder vaccination in patients receiving Anti-TNF.

Theme	Sub-theme	Supporting quote
**Physician**	Knowledge	*“Lack of knowledge*, *sometimes*, *lack of awareness about the importance of vaccine in our patient*.*” (P5)*
	Education	*“I think both physician education*, *or they know*, *but maybe sometime they forget” (P10)*
	Awareness	*“Number one is physician awareness*, *and this is very important*. *Awareness doesn't mean that they only know*, *but I mean*, *that they should be much aware of the importance much*.*” (P1)*
	Belief	*“The main barrier because most of the dermatologists they think that the dose that they are using is dosing*, *which is not the same as other specialties*. *It's more of immunomodulation*, *not immunosuppression*, *so why to give vaccination to patient who is low risk*?*” (P2)*
**Patient**	Awareness	*“I think patient awareness*.*” (P10)*
	Disease State	*“Other point*, *maybe*, *because sometimes our patients are so sick*. *As I mentioned to you*, *patients sometimes not give you a chance to looking for everything*.*” (P5)*
	Education	*“I think patient education as well*. *It is important that patient*, *they know*. *Sometime patient will remind you as well*.*”(P10)*
	Beliefs	*“Some patients refuse to take vaccines*. *They think vaccines can cause themselves disease” (P7)*
	Convenience	*“All of us know that*, *yes*, *we know not to give the vaccinations at least 2 weeks prior*, *but the problem I face myself is to time it*, *because once we see the patient today*, *for example*, *and I decide on starting Anti-TNF therapy*. *I cannot ask the patient to come back only for the vaccine*, *giving it a two-week time*, *and then resume it*.*” (P3)*
**Health Care System**	Electronic system	*“Now*, *with an electronic prescription*, *and this checklist (vaccination recommendations) is not uploaded in the system of e-sihi So*, *I think the adherence (to vaccines)*, *it's not so good*.*” (P4)*
	Other HCPs’ Role	*“I think from the pharmacy side if anything to link (vaccination recommendations) to the (electronic system in the hospital) so we make adherence will be even great*.*” (P4)*
	Workload	*“You know clinics are very busy clinics and then we are mainly concentrating on when giving the patient Anti-TNFs*, *in the response*, *not preventing side-effects*.*” (P1)*
	Policy implementation	*“Guideline is there* .* *.* *.*now they have written protocol*, *maybe they are* .* *.* *. *(guidelines) should be implemented practically*, *in our daily practice*.*” (P10)*
	Vaccines availability	*“Availability of the vaccine*. *The availability of the vaccine*. *Sometimes it's not available*.*” (P8)*

## Discussion

This study is considered the first to use a mixed-methods approach to explore the use of vaccines among patients who were prescribed Anti-TNF therapy and variations among physicians’ behavior that affect vaccine administration. The results highlighted very low vaccine use and variations in awareness, adherence, practice, and perception among physicians as well as the main barriers that prevent them from adhering to the recommendations.

A few quantitative studies have addressed adherence to vaccination recommendations when prescribing Anti-TNF therapy; most concluded that vaccine use was suboptimal [30–39]. The percentage of vaccinated patients in our study was relatively low (13.9%), compared to that in a study conducted by Malhi et al. in 2015, which concluded that 41.2% of its patients had been vaccinated with 61.3%, 61.0%, 20.7%, and 10.3% of their IBD patients completing influenza, hepatitis B, meningococcal, and pneumococcal vaccines, respectively, while on Anti-TNF therapy [35]. Other similar studies have discussed the vaccination rates of individual vaccines. In 2012, Feuchtenberger et al. reported that 69.2% and 36.8% of RA patients who received Anti-TNF therapy also received influenza and pneumococcal vaccines, respectively (31). Another study conducted by Krasselt et al. (2016) showed that 53% and 33% of RA patients received influenza and pneumococcal vaccines [33]. The highest vaccination rate was reported by Wasan et al. in 2014; 81.5% and 42.6% of IBD patients in a setting in the US received influenza and pneumococcal vaccines, respectively, while receiving Anti-TNF therapy (38). In a study by Sandler et al. (2016), which had a high vaccination rate, 79.4% of the study sample reported receiving the influenza vaccine while 53.9% reported receiving the pneumococcal vaccine [37]. Hua et al. (2015) reported vaccination rates of 53% and 59.7% for influenza and pneumococcal vaccines, respectively [32]. Possible explanations for these relatively high vaccination rates could be the use of self-report as a method of data collection, the large sample size, or awareness of the vaccination recommendations in the study sample. One of the first studies to investigate the use of vaccines in IBD population stated that the use of the influenza vaccine was about 28% while that of the pneumococcal vaccine was less than 10% [36]. These results are considered lower than that reported in our study; the most common reasons for these lower results were due to lack of awareness and concern for side effects. Our study had good adherence to vaccination recommendations; however, the vaccination rate cannot be labeled as optimal.

Nevertheless, three studies reported vaccination rates relatively lower than those reported in our study while patients were treated with Anti-TNF therapy. The first study was conducted by Luque et al. in 2016 and reported vaccination rates of 13.2% and 15% for influenza and pneumococcal vaccines, respectively, in RA patients. Another study conducted in 2017 by Bonhomme et al. reported corresponding rates of 13.2% and 4.4% in psoriasis patients [30, 34]. The third study was conducted by Wilckens et al. in 2011 and reported vaccination rates of 19% and 3% for influenza and pneumococcal vaccines, respectively, in IBD patients [39]. Apart from the study by Malhi et al. (2015), no study has reported the HBV vaccination status of the sample population [35]. Because the risk of contracting or reactivating HBV during Anti-TNF therapy is high, the guidelines recommend complete vaccination for HBV in patients who have never been vaccinated before or a booster dose of HBV vaccine in patients who were vaccinated for it during childhood, before initiating Anti-TNF therapy [7, 23–29]. The variability in the reported vaccination rates clearly indicates variation in physicians’ practice, thus justifying this qualitative study aimed at exploring this phenomenon.

The qualitative study findings showed evident variations in opinions among physicians with regard to the four main categories of description, namely awareness, adherence, practice, and perception. This may explain the low vaccination status observed in the quantitative results. Most of the physicians were aware of the existence of specific vaccination recommendations for those prescribed Anti-TNF therapy. However, this claimed awareness did not quite translate into adherence to these recommendations. The physicians who stated that they adhere to the vaccination recommendations were found to be only from the gastroenterology department, which explains the fact that gastroenterologists prescribed vaccines the most compared to other physicians. The high percentage of vaccinations attributed to the pulmonology department was not due to the physicians’ knowledge of or adherence to the vaccination recommendations for those prescribed Anti-TNF therapy, but rather due to the common practice of vaccinating all patients who are identified with a respiratory disease. During the interview, one of the pulmonologists stated, *“Before (Anti-TNF) initiation*, *we assess the patient’s schedule for vaccination because most patients*, *regardless of whether we are giving them infliximab or not*, *are keen to start the pneumococcal and influenza vaccines*. *This is part of the pulmonary disease protocol*, *and not limited to infliximab”* (P11).

The findings of the phenomenographic analysis showed that most of the physicians from the dermatology department claimed to vaccinate certain patients only, since they believe that use of vaccines depends on patients’ risk of infection and that the Anti-TNF doses they use are non-immunosuppressant in nature. One dermatologist said, *“The dose (of Anti-TNF) that we are using is different from those used in other specialties*. *It is more likely to immunomodulate*, *not to immunosuppress; so why give it (vaccines) to a patient who is at low risk (of infection)*?*”* (P2). Such perceptions are reflected in the fact that none of the vaccines issued to the patients in this study were from the dermatology department.

Most of the physicians admitted not adhering to vaccination recommendations and attributed this behavior to multiple factors, as evident in the thematic analysis. These factors were broadly categorized into physician-related barriers, patient-related barriers, and healthcare-system-related barriers.

When the thematic analysis findings regarding physician-related barriers were explored, more than half of the participants identified that a lack of knowledge or awareness was the major contributor to this category of barriers and responded, *“A lack of knowledge*, *sometimes a lack of awareness about the importance of vaccines for our patients”* (P5). Similarly, two previous quantitative studies, one of which was conducted in the US, concluded that a lack of knowledge was the reason for physicians’ poor vaccination recommendations in IBD patients [42, 43].

As for the patient-related barrier, the physicians believed that by the time patients reach the stage when they go to the clinic, they are already very sick and demand immediate treatment to control their disease and responded, *“Maybe because sometimes our patients are so sick*. *As I mentioned to you*, *patients sometimes don’t give you the chance to look for everything”* (P5). Additionally, patients’ beliefs and concerns about vaccine safety were also highlighted by the physicians participating in this study, *“Some patients refuse vaccines*. *They think vaccines themselves can cause disease”* (P7). This barrier was also identified by Malhi et al. (2015) and Melmed et al. (2006) in their studies, which were conducted in the US and Canada, as reasons behind suboptimal vaccine uptake [35, 36].

Also for the healthcare-system-related barriers, the physicians strongly believed that other healthcare professionals such as nurses, pharmacists, and family medicine physicians should be engaged to improve vaccination uptake. One of the rheumatologists stated, *“Definitely*, *we need a multi-disciplinary effort*. *If nurses are trained more about vaccines and protocol*, *it would help because*, *as physicians*, *we are busy with other things*. *I think if a rheumatology nurse is trained in this*, *it will be a good option for them”* (P10). One of the gastroenterologists agreed, *“I think*, *from the pharmacy side*, *if anything*, *we need to link (vaccination recommendations) to the system so that adherence (to vaccination recommendations) increases further”* (P4). This finding is in line with the results from a quantitative study conducted in 2015 in Canada by Malhi et al., which demonstrated that the annual vaccination reviews of IBD patients by family physicians (OR = 1.82) or gastroenterologists (OR = 1.72) were significant predictors of vaccine completion. The majority of their patients stated that the primary responsibility for ensuring vaccine completion lies with the patient (41.7%) and the family physicians (32.3%) [35]. Another study by McCarthy et al. (2012), focused on practicing Irish rheumatologists, showed that vaccination is the role and responsibility of general practitioners [44]. Gupta et al. (2011) stated that many IBD specialized physicians do not recommend vaccines at all to their patients; they also believed that ensuring vaccination status is a shared responsibility between specialists and general practitioners [45]. At our hospital, although clinical pharmacists regularly remind physicians to vaccinate their patients who are starting Anti-TNF therapy, especially in the rheumatology department, this does not improve their practice, as shown in Table 3. Another important barrier is policy implementation: *“Guidelines are there; now there is a written protocol; they should be implemented in our daily practice”* (P10).

Interestingly, some of the participants brought physicians’ workload into the discussion, considering it one of the main healthcare-system-related barriers: *“You know we have very busy clinics and we mainly concentrate on when to give patients Anti-TNF therapy and on looking for a response*, *not on preventing side-effects”* (P1). Another interesting barrier we found was vaccine availability, highlighted by 25% of the physicians: *“Sometimes vaccines are not available”* (P8). The physicians in this study identified these two barriers as major influences on vaccine uptake; however, these barriers have not been discussed by other studies.

In summary, using the mixed-methods approach highlighted the fact that even though most of the physicians in our study recognized the importance and benefits of using vaccines and claimed that they prescribe them in their practice, the quantitative results contradict their claims and perception; only 13.9% of the patients on Anti-TNF therapy received any sort of prior vaccination.

To improve the observed low vaccination uptake, several promising tools that have been suggested in the literature can be applied. In 2016, Corace et al. identified psychological theories of behavior change as potential tools to improve influenza vaccination uptake among healthcare workers [46]. A study by Walsh et al. (2013) of the implementation of a screening and vaccination pro forma showed that self-reported gastroenterologist screening for vaccination notably improved from 47% to 97% before and after the intervention, respectively, and also increased the vaccination against HBV, varicella, influenza, and *Pneumococcus* infection. However, their patients’ compliance with the vaccination did not significantly improve; therefore, they suggested exploring different strategies to enhance patient education [47]. Another study reviewed personal health reports by clinical pharmacists who had never vaccinated for herpes zosters before and found that reminding patients by electronic message or by mail significantly improved vaccine use compared to that associated with standard care [48]. Integrating patients’ vaccination status into a health information system that produces pop-up messages or reminders is also a potential tool for enhancing vaccination uptake and is thus worth investigating.

This study has several strengths, such as the use of the mixed-methods approach. This method helped not only to identify the extent of the problem but also to explore and explain the behavior behind the phenomenon. From the methodology perspective, use of data derived from the EMRs instead of surveys helped avoid the recall bias that may have affected the results of similar studies and allowed the use of a large sample size. Multiple measures were used to ensure the trustworthiness of the qualitative study, maintaining credibility, transferability, dependability, and confirmability, and thus building the strength of the study as a whole. These measures included approaching physicians with different specialties to maximize variation, gathering background knowledge on vaccination, using an interview guide, using introductory questions, ensuring the consistency of transcripts by involving two independent researchers, and presenting findings by using appropriate quotations. Triangulation was maintained to ensure reliability by using two electronic recorders, two interviewers, and two methods of data analysis.

The limitations of the study are as follows. The lack of other similar qualitative or mixed-methods studies limited the comparison of our findings with other reported findings. Notably, the use of an electronic data source in the quantitative study led to issues of data incompleteness and missing entries. To overcome these problems, manual checking and data extraction were performed to ensure data completeness. Another possible limitation of this study can be related to the generalizability of qualitative findings of this research. To overcome this limitation and strengthen the generalizability of these findings, thick description, which include a comprehensive description of the research method and data interpretation process, and an in-depth presentation of the findings were used. Moreover, the use of mixed-methods approach by itself hold promise for generalizability.

## Conclusions

The use of Anti-TNF therapy poses an infection risk to patients and vaccination is required before initiating the therapy; nevertheless, the vaccination rate remains low. We have identified variation in physicians’ behavior and barriers, similar to those identified in studies conducted in the US that determine such a phenomenon. Hospitals must overcome these barriers by developing policies, procedures, and protocols for vaccination, by introducing the pro forma for vaccination, by implementing education programs for both physicians and patients, and by ensuring an appropriate procurement of vaccines. Future research could be conducted to compare the cost of vaccination use and the cost of infection arising from a lack of proper vaccination. This would help decision-makers to understand the importance of enforcement of vaccine recommendations in such patients. Implementation research could also be performed to investigate the effect on vaccination status of any intervention that alters the behavior of either the physicians or the patients who are prescribed Anti-TNF therapy.

## Supporting information

S1 File Interview topic guide. (DOCX)
