# Supplementary material for: Vaccination status of patients using anti-TNF therapy and the physicians’ behavior shaping the phenomenon: Mixed-methods approach
Source: PLoS One. 2019 Oct 4;14(10):e0223594. doi: 10.1371/journal.pone.0223594 (PMC6777782; doi:10.1371/journal.pone.0223594)
Supplement: S1 File — (DOCX) [file pone.0223594.s001.docx]

**INTERVIEW TOPIC GUIDE**

**The following are the questions that make up the interview guide:**

- 1. What are your perceptions of Anti-TNFs as medications?
  2. What are your views regarding Anti-TNFs prescribing in general?
  3. In your daily practice, how often do you use and follow Anti-TNFs treatment recommendations or guidelines?
  4. When you decide to initiate an Anti-TNF, what are the recommendation(s), guideline(s), or protocol(s) that you follow to screen the patient before Anti-TNF administration?
  5. What is/are the name of the recommendation(s) and/or guideline(s) that you follow?
  6. What are your perceptions of vaccine use with Anti-TNFs therapy?
  7. Are you aware of any vaccination recommendation(s) and/or guideline(s) associated with the administration of Anti-TNFs? If yes, then which ones and when was the last time you reviewed them?
  8. Do you use and follow vaccination recommendation(s) and/or guideline(s) associated with the administration of Anti-TNFs in your daily practice? If yes, how often?
  9. In your view, what do you believe to be the gaps, factors or barriers that are influencing physicians’ adherence to vaccination recommendation(s) and/or guideline(s) with Anti-TNFs use?
  10. Is there anything else you think I have missed in this interview that you would like to add?

**The followings are generic probing and prompting questions that may be used in the interviews with physicians:**

1. Could you explain that further?

2. Could you please give me an example?

3. What do you mean by that?

4. Can you tell me more about that?

5. Can you explain that in a different way?

6. Is there anything else you would like to say about this?
